# Supplementary material for: Inwardly rectifying potassium channels promote directional sensing during neutrophil chemotaxis
Source: J Cell Biol. 2025 Nov 19;225(1):e202503037. doi: 10.1083/jcb.202503037 (PMC12629209; doi:10.1083/jcb.202503037)
Supplement: SourceData F5 — is the source file for Fig. 5. [file jcb_202503037_sourcedataf5.pdf]

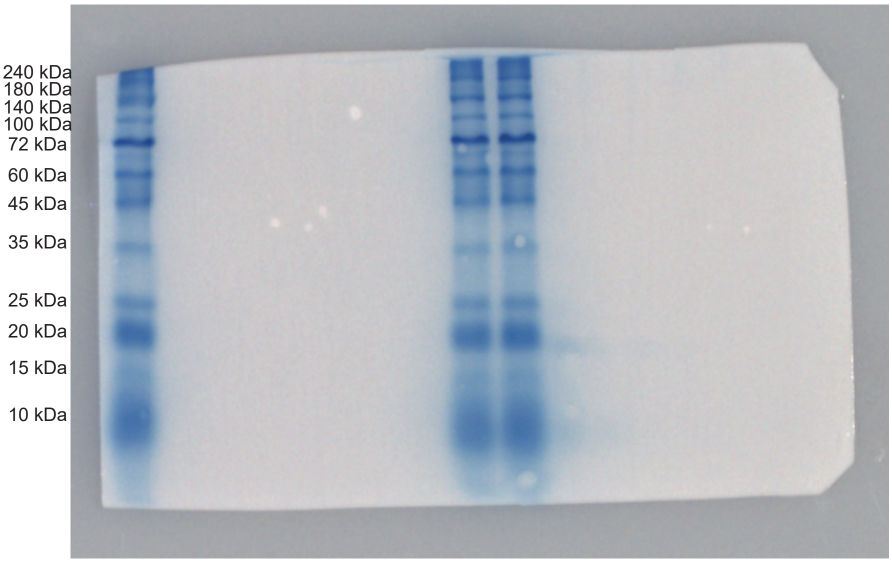
240 kDa
180 kDa
140 kDa
100 kDa
72 kDa
60 kDa
45 kDa
35 kDa
25 kDa
20 kDa
15 kDa
10 kDa

p-PAK

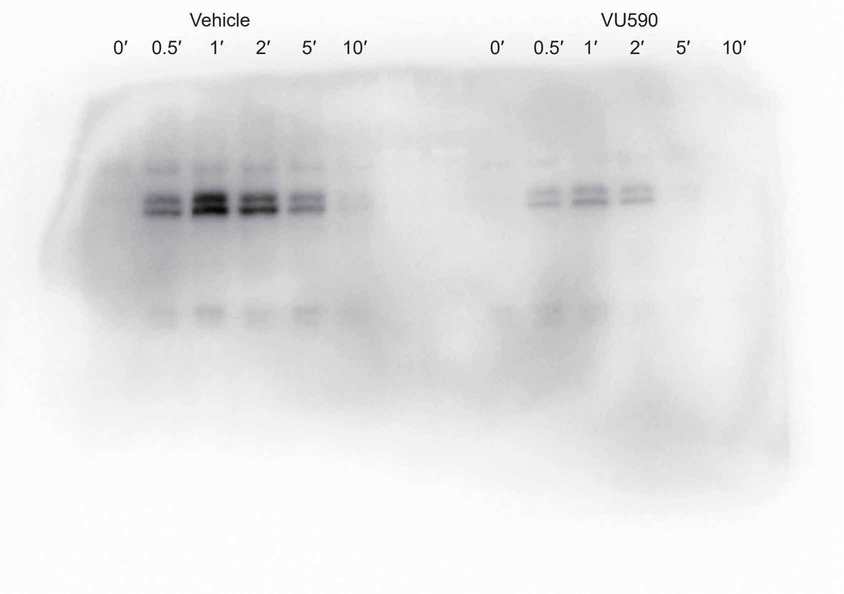

p-PAK

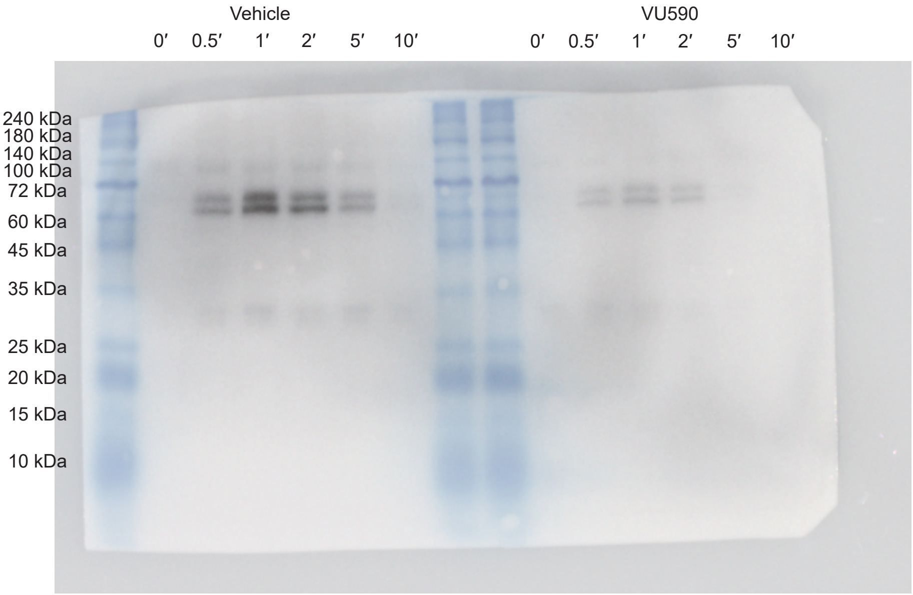
Vehicle
VU590
0′ 0.5′ 1′ 2′ 5′ 10′
0′ 0.5′ 1′ 2′ 5′ 10′
240 kDa
180 kDa
140 kDa
100 kDa
72 kDa
60 kDa
45 kDa
35 kDa
25 kDa
20 kDa
15 kDa
10 kDa

PAK123

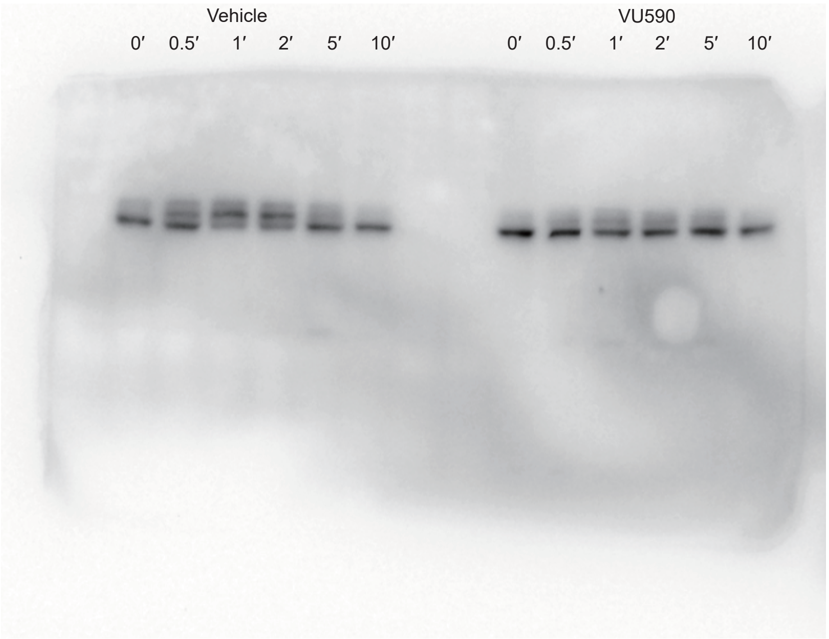

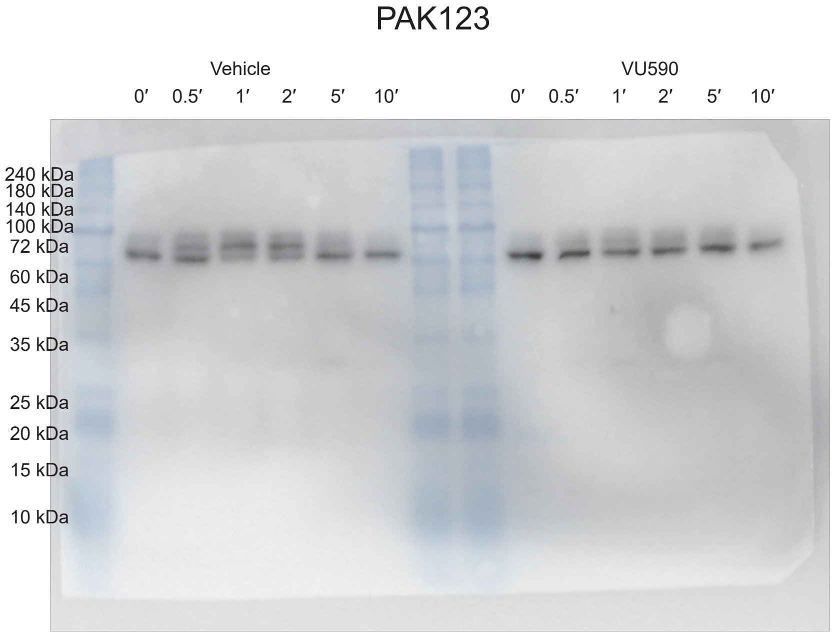
PAK123
Vehicle
VU590
0′
0.5′
1′
2′
5′
10′
0′
0.5′
1′
2′
5′
10′
240 kDa
180 kDa
140 kDa
100 kDa
72 kDa
60 kDa
45 kDa
35 kDa
25 kDa
20 kDa
15 kDa
10 kDa

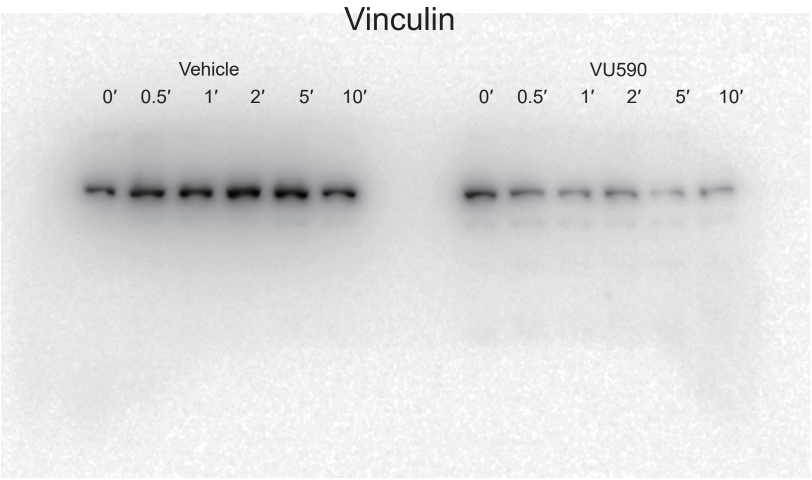
Vinculin
Vehicle
VU590
0′ 0.5′ 1′ 2′ 5′ 10′
0′ 0.5′ 1′ 2′ 5′ 10′

Vinculin

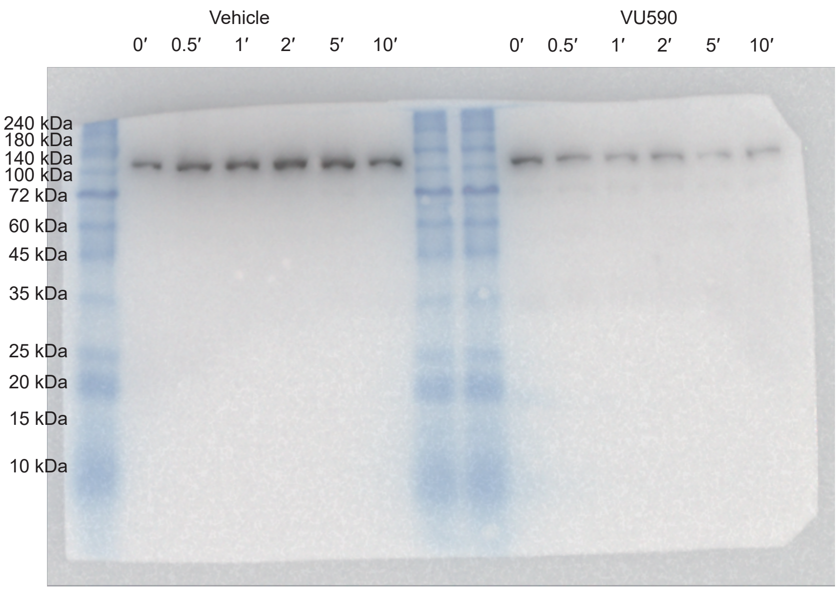
Vehicle
VU590
0′
0.5′
1′
2′
5′
10′
0′
0.5′
1′
2′
5′
10′
240 kDa
180 kDa
140 kDa
100 kDa
72 kDa
60 kDa
45 kDa
35 kDa
25 kDa
20 kDa
15 kDa
10 kDa

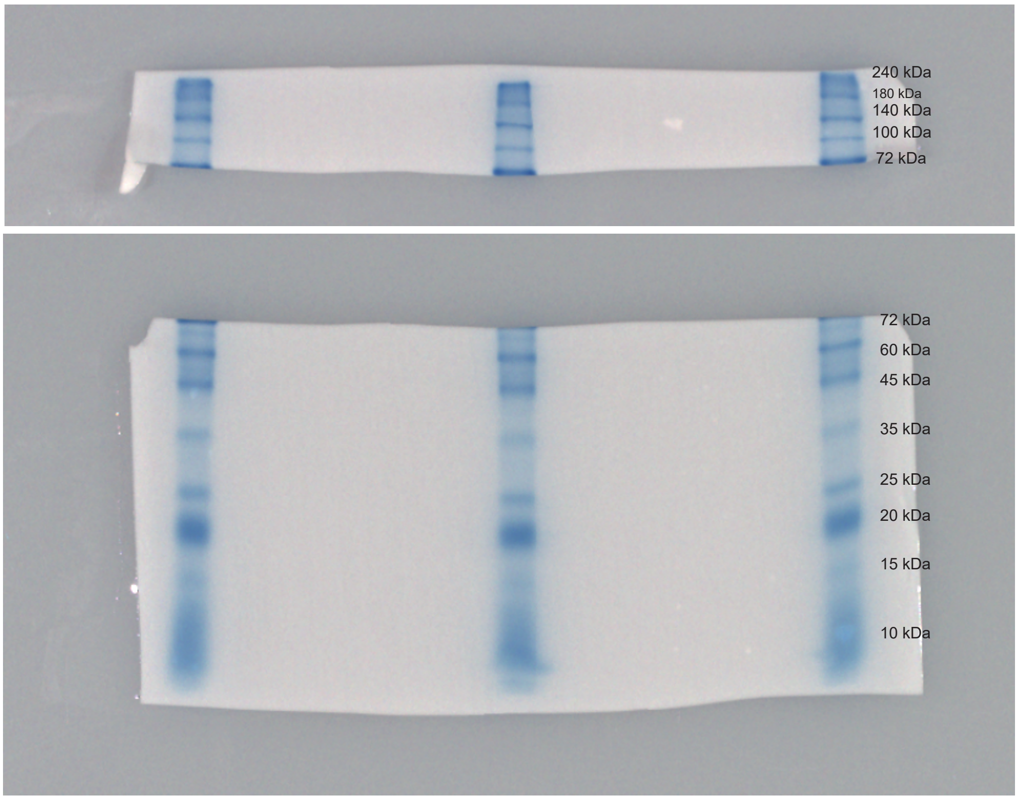
240 kDa
180 kDa
140 kDa
100 kDa
72 kDa
72 kDa
60 kDa
45 kDa
35 kDa
25 kDa
20 kDa
15 kDa
10 kDa

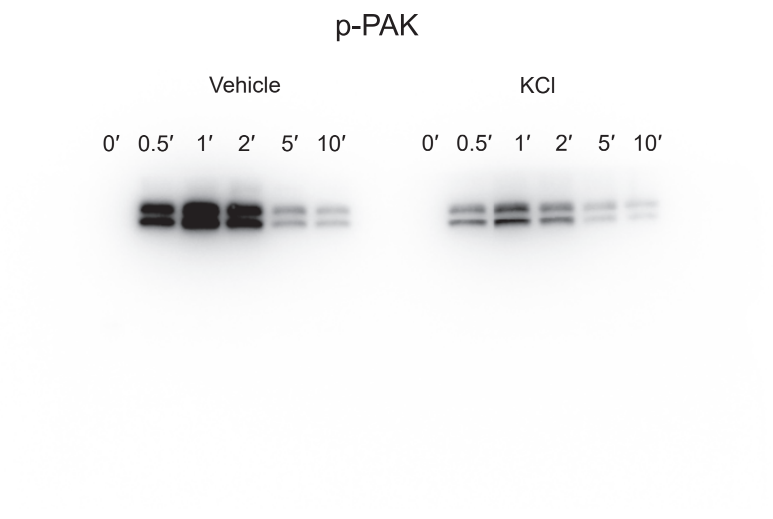
p-PAK
Vehicle
KCl
0′ 0.5′ 1′ 2′ 5′ 10′
0′ 0.5′ 1′ 2′ 5′ 10′

p-PAK

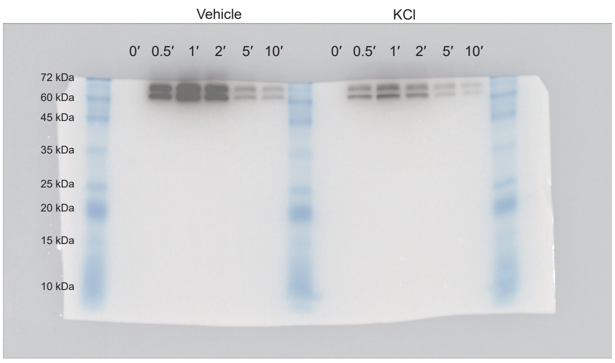
Vehicle
KCl
0′ 0.5′ 1′ 2′ 5′ 10′
0′ 0.5′ 1′ 2′ 5′ 10′
72 kDa
60 kDa
45 kDa
35 kDa
25 kDa
20 kDa
15 kDa
10 kDa

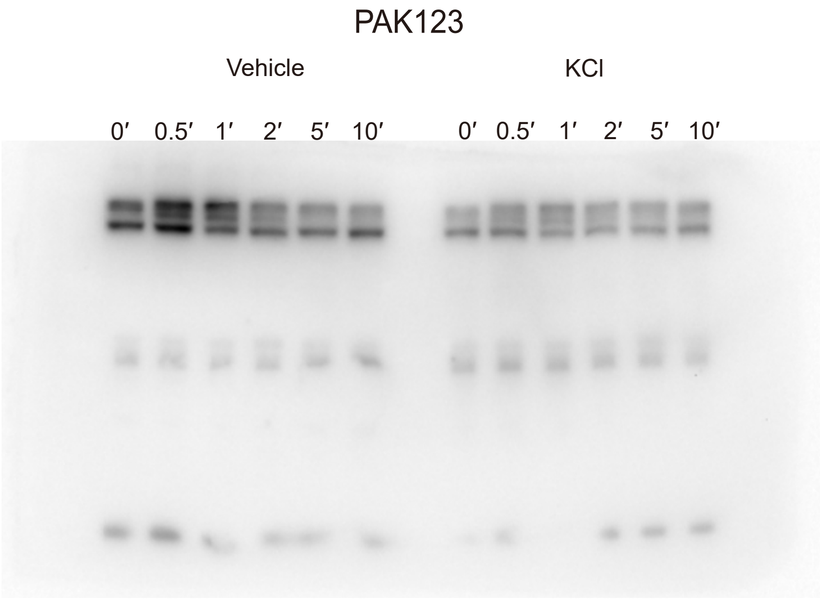
PAK123
Vehicle
KCl
0′ 0.5′ 1′ 2′ 5′ 10′
0′ 0.5′ 1′ 2′ 5′ 10′

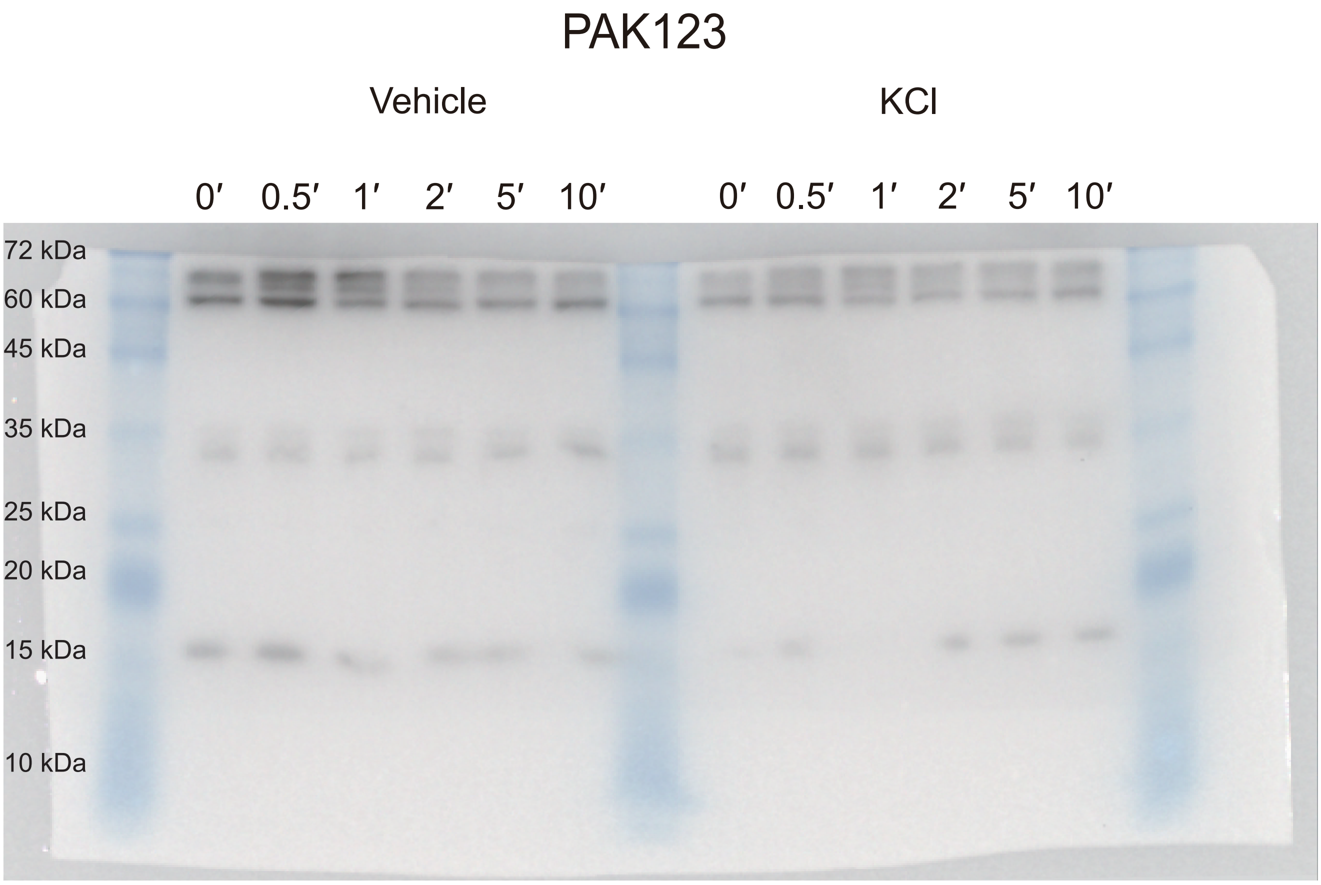
PAK123
Vehicle
KCl
0′ 0.5′ 1′ 2′ 5′ 10′
0′ 0.5′ 1′ 2′ 5′ 10′
72 kDa
60 kDa
45 kDa
35 kDa
25 kDa
20 kDa
15 kDa
10 kDa

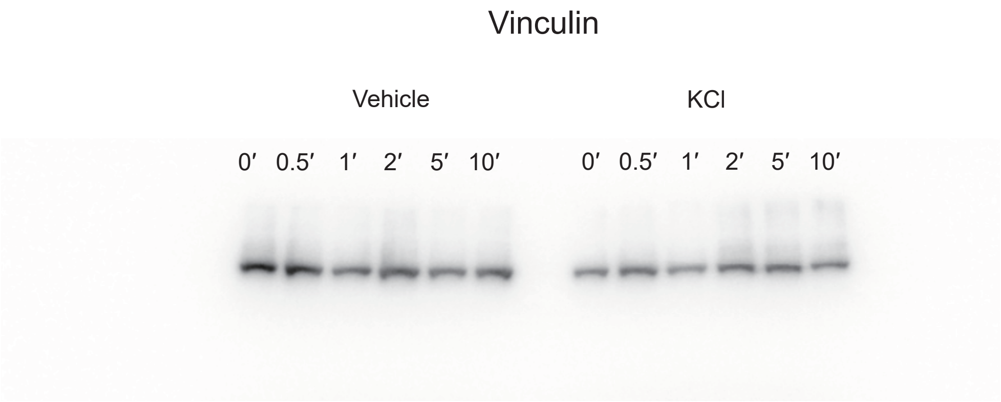
Vinculin
Vehicle
KCl
0′ 0.5′ 1′ 2′ 5′ 10′
0′ 0.5′ 1′ 2′ 5′ 10′

Vinculin

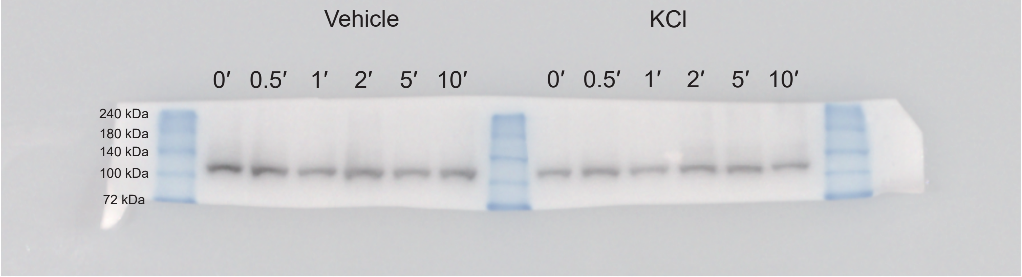
Vehicle
KCl
0′ 0.5′ 1′ 2′ 5′ 10′
0′ 0.5′ 1′ 2′ 5′ 10′
240 kDa
180 kDa
140 kDa
100 kDa
72 kDa
