# Supplementary figures and images for: Inwardly rectifying potassium channels promote directional sensing during neutrophil chemotaxis
Source: J Cell Biol. 2025 Nov 19;225(1):e202503037. doi: 10.1083/jcb.202503037 (PMC12629209; doi:10.1083/jcb.202503037)

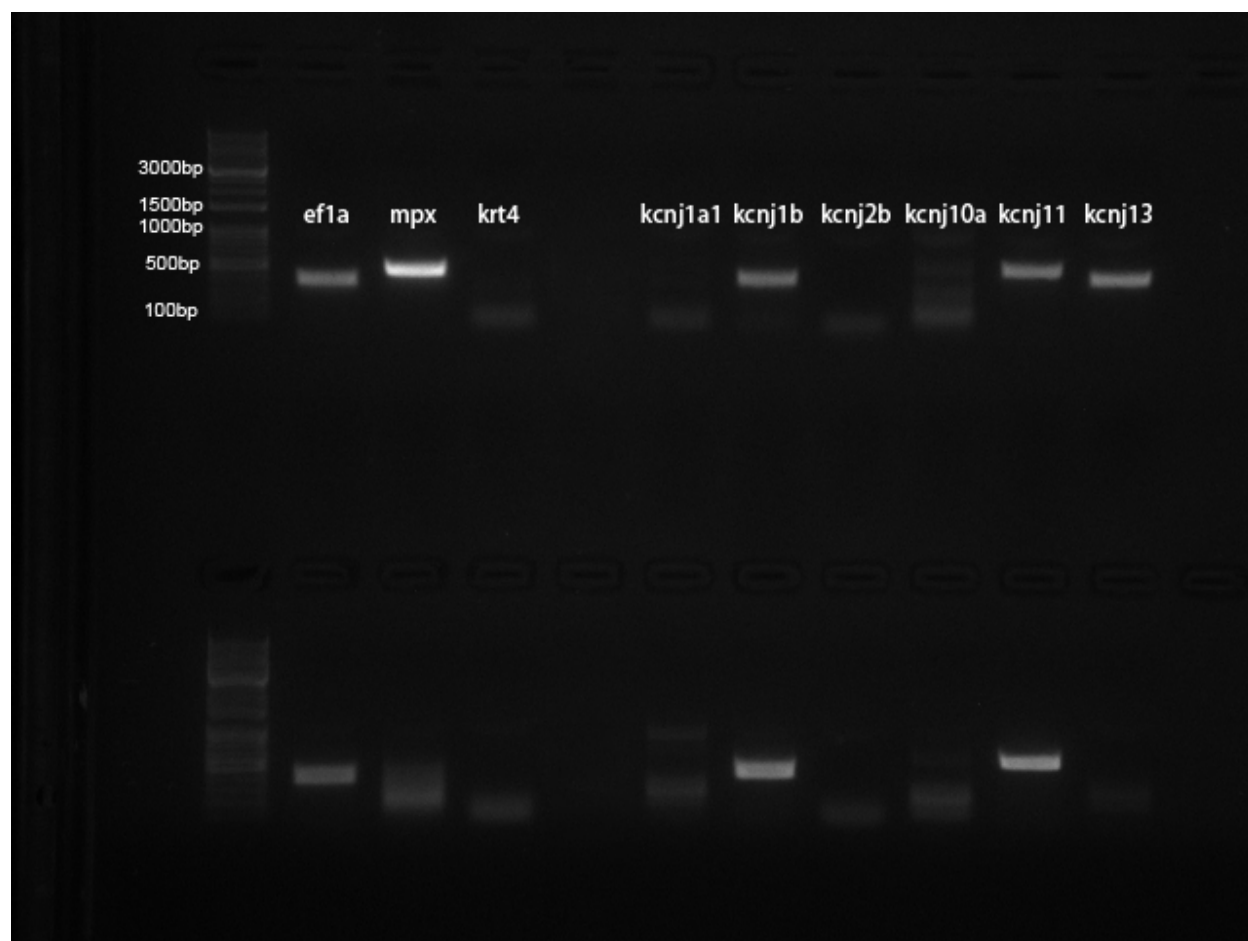

Supplement: SourceData FS1 — is the source file for Fig. S1. [file jcb_202503037_sourcedatafs1.pdf]
